# NiO/Ni Nanowafer Aerogel Electrodes for High Performance Supercapacitors

**DOI:** 10.3390/nano12213813

**Published:** 2022-10-28

**Authors:** Ramya Ramkumar, Ganesh Dhakal, Jae-Jin Shim, Woo Kyoung Kim

**Affiliations:** School of Chemical Engineering, Yeungnam University, Gyeongsan 38541, Korea

**Keywords:** supercapacitor, metal aerogels, porosity, surface area, energy storage

## Abstract

Transition metal oxide aerogels are a fascinating class of compounds that have received considerable attention in the last decade owing to their unique and exceptional properties, including high porosity, large surface area, and ultralow density. In this study, α-Ni(OH)_2_ aerogels and annealed NiO/Ni aerogels were used to design and fabricate a two-electrode supercapacitor device. The physicochemical properties of the as-synthesized aerogels were characterized through X-ray diffraction, scanning electron microscopy, transmission electron microscopy, the Brunauer–Emmett–Teller theory, and X-ray photoelectron spectroscopy studies. The annealed NiO/Ni aerogels showed a (specific capacitance of 1060 F/g) specific capacity of 422 C/g at 1 A/g current density and with good cycling stability (up to 10,000 cycles). The supercapacitor also demonstrated an energy density of 32.4 Wh/kg and power density of 1800 W/kg at a current density of 2 A/g. The specific capacitance of NiO/Ni aerogels was more than twice that of the α-Ni(OH)_2_ aerogels. The practical applications of the aerogel were demonstrated by fabricating a two-electrode device.

## 1. Introduction

Electrochemical supercapacitors with high power density (PD), good reversibility, wide thermal operation range, and good cycling stability have emerged as promising candidates for use in hybrid electric vehicles, regenerative braking, uninterruptible power supply (UPS), and solar power applications [1]. In general, supercapacitors have been employed as replacements for conventional vehicle batteries, where they are connected across a small lead–acid battery. However, despite these advantages, supercapacitors lack energy density (ED), which restricts their practical applications. To overcome this shortcoming, hybrid supercapacitors have been developed, which have high ED and good cycling stability [2]. The positive and negative electrodes of a hybrid two-electrode supercapacitor are made of a pseudocapacitive material and carbon, respectively. Such devices exhibit outstanding properties, including high PD, very fast charging/discharging rates, excellent cycling stability, and low manufacturing costs [3,4,5].

The nature of an electrode material determines its performance. Various electrode materials, such as Ni_3_S_2_/NiV [6], BFCNi [7], Ni-NiO [8], Ni_3_S_4_ [9], NiO/Ni [10], and Ni-Co [11], have been explored for supercapacitor applications. The reported energy materials have exhibited specific capacitance with good cycling stability. However, the energy density exhibited in some iron and carbon based materials ranges from 17.8 to 88 Wh/kg, and their power densities vary from 652, 800, to 8000 W/kg for porous carbon [12], FeCo_2_S_4_/rGO [13], and Fe-Co-Ss [14], respectively. Various other metal oxide- and polymer-based supercapacitors include MnO_2_based stretchable nanowires [15], PANI/BC-based [16] supercapacitors, and Ni metal ions inside carbon micropores [17], which also have exhibited useful practical applications. For the better performance of a supercapacitor, the main criteria are good porosity, uniform structure, and abundant active sites. Aerogels, as active materials, play a key role in energy storage devices because of their ultralow densities, high surface areas, and excellent porosities [18]. Many researchers have attempted to combine the effects of electric double-layer capacitors (EDLCs) [19] and pseudocapacitive [20]-based electrode materials to enhance the performance of hybrid devices. However, EDLC-based pure graphene aerogels exhibit a low specific capacitance of 200 F/g [21]. To meet increasing energy demands, we need to search for active materials that have a higher specific capacitance with excellent cycling stability and low weight. In this regard, several research studies on graphene aerogels for supercapacitor applications, either individually [21] or with metal nanoparticles [22] or oxides [23], have been conducted in detail.

Among the different materials used for supercapacitor applications, we explored the pseudocapacitive nature of novel metal oxide aerogels in this study. The difference between nanoparticles and aerogels is that the latter have low mass density, high porosity, and a very large specific surface area, which are cost-effective and the most desirable features for energy storage applications. Among many transition metals, nickel has been shown to exhibit unique properties, such as superior ferromagnetic nature, chemical stability, cost-effectiveness, and abundance in the Earth’s crust [24]. NiO materials exhibit excellent physical as well as chemical properties such as mesoporous hierarchical porous structures, high surface area, and increased electronic conductivity. NiO nanoparticles exhibit p-type semiconductivity with a band gap ranging from 3.6 to 4.0 eV [25]. Owing to their enhanced activity and superior physicochemical properties, NiO particles are used as electrode materials for supercapacitor applications, in addition to various other applications, such as catalysts, active materials in Li-ion batteries, removal of heavy metals from wastewater, electronic devices, and sensors [26,27,28]. The flaky NiO on stainless steel substrates obtained via electrochemical synthesis has exhibited good capacitance and has been employed in supercapacitor electrodes [29]. The practical capacitance obtainable by nickel-based materials is around 1000 F/g, and the theoretical capacitance of NiO is estimated to be 3750 F/g, making it a good material that exhibits batterylike behavior in supercapacitors. Thus far, several researchers have worked on nickel on silica or graphene aerogels for various applications by depositing, incorporating, or doping nickel nanoparticles onto silica aerogel frameworks [30,31].

In the present study, for the first time, we synthesized α-Ni(OH)_2_ hydrogels and converted them into aerogels using a freeze-drying technique. The prepared α-Ni(OH)_2_ aerogels were annealed at 400 °C to form NiO/Ni aerogels with good porosity and networklike continuous structures composed of nanoflakes of Ni moiety. The enhanced performance of NiO/Ni aerogels over α-Ni(OH)_2_ aerogels in terms of supercapacitive behavior was investigated in detail in this study. The significance of this study involves a simple synthesis method, a single ingredient, and the instant formation of hydrogels. Using activated carbon (AC) as the negative electrode and NiO/Ni as the positive electrode, a two-electrode device was fabricated to evaluate this aerogel’s practical applications. In terms of PD and cycling stability, the device performed very well. This study paves the way for lightweight and easy-to-handle supercapacitors for industrial, automotive, and aeronautic applications.

## 2. Experimental Study

### 2.1. Materials

Nickel nitrate hexahydrate (Ni(NO_3_)_2_·6H_2_O, 99%), sodium borohydride (NaBH_4,_ 99%), and sodium sulfate (Na_2_SO_4_, 99%) were obtained from Sigma Aldrich (St. Louis, MO, USA). Ethanol and acetone were purchased from Alfa Aesar (Haverhill, MA, USA). The nitrogen gas cylinder was purchased from a local dealer (Gyeongsan, Korea). All experiments were performed with deionized water, and the temperature was maintained at 25 °C, unless otherwise specified.

### 2.2. Preparation of Ni and NiO Aerogels

The protocol to prepare the transition metal aerogels was adopted from a previous synthesis procedure [18]. Ni(NO_3_)_2_·6H_2_O was completely dissolved in a mixture of 25 mL ETOH + 75 mL of deionized (DI) water (total volume: 100 mL). Nitrogen gas was bubbled through the salt solution for 25 min. Next, 100 mL of 0.5 M NaBH_4_ solution was prepared by dissolving it in ethanol and placing it in a standard flask. The NaBH_4_ solution was added to the nickel nitrate hexahydrate solution. Immediately, the solution turned black, indicating that α-Ni(OH)_2_ hydrogels had formed. The as-formed hydrogels floated to the top of the solution because of bubbles arising from the NaBH_4_ solution. The reaction was allowed to proceed for 5 min. Next, the precipitate was washed 5–6 times with a solution of DI water and ethanol. The black hydrogel obtained was dispersed in DI water until the precipitate was immersed. The samples were freeze-dried for two days. This sample was designated as the Ni aerogels and stored in a desiccator for further use. Subsequently, some of the as-formed Ni aerogels were annealed in air at 400 °C at a heating rate of 10 °C/min for 2 h to form NiO aerogels, which were stored in a desiccator for further use.

### 2.3. Characterizations

The morphological, structural, and physical properties of the as-formed aerogels were analyzed using measurement techniques, such as X-ray diffraction (XRD; X’Pert PRO MPD, Malvern Panalytical, Almelo, Netherlands), Fourier transform infrared (FTIR; Spectrum100, PerkinElmer, Waltham, MA, USA) spectroscopy, X-ray photoelectron spectroscopy (XPS; ESCALAB 250, Thermo Fisher Scientific, Altrincham, UK), high-resolution scanning electron microscopy (HR-SEM; S-4800, Hitachi, Tokyo, Japan), high-resolution transmission electron microscopy (HR-TEM; FEI Tecnai G2 F20, acceleration voltage-200 kV, Barcelon, Spain), and the Brunauer–Emmett–Teller (BET; 3Flex Version 3.02, Micromeritics, Atlanta, GA, USA) theory. Using an Autolab PGSTAT302N (Metrohm, Utrecht, The Netherlands), all electrochemical studies, including electrochemical impedance spectroscopy (EIS), cyclic voltammetry (CV), and galvanostatic charge–discharge (GCD), were conducted. The 3M KOH electrolyte was used for all the electrochemical measurements. The working electrode was the modified Ni foam (size: 1 cm × 1 cm; loading: ~2 mg), whereas the reference electrode was either a silver/silver chloride (Ag/AgCl) electrode or a saturated calomel electrode (SCE). The Pt foil was used as the counter electrode (BASi, West Lafayette, IN, USA) for a three-electrode arrangement. The working electrodes were made by combining the α-Ni(OH)_2_ or NiO aerogel (85%), carbon black (10%), and PVDF (poly(vinylidene fluoride)) (5%), and N-methyl pyrrolidone (NMP) as a mixing solvent to obtain a slurry. The slurry was coated onto a Ni foam (NF) and then dried at 60 °C for 12 h.

### 2.4. Electrochemical Measurements for NiO/Ni/NF Electrode

The CV measurements were obtained in the potential window of 0 to 0.55 V vs. Ag/AgCl (filled with saturated KCl) using scan rates of 2 to 50 mV/s. GCD measurements were obtained at current densities from 1 to 15 A/g in the selected potential range of 0 to 0.4 V vs. Ag/AgCl. All electrochemical impedance spectroscopic (EIS) measurements were obtained at an open-circuit voltage within a frequency range of 10 mHz to 10 kHz and an amplitude of 5 mV.

The voltammetric specific capacitance (*Cs*, F/g) was calculated using the following equation [32]:

(1) $$C_{s}=\frac{\oint i\times dV}{VS\times m}$$

where ∮*idV*(in A.V) is the voltammetric charge represents the area under the curve in voltammetry, *V* is the potential window (in V), *S* is the scan rate (in V/s), and *m* is the mass loading of the active material (in g) on the Ni foam working electrode.

### 2.5. Fabrication of the NiO/Ni/NF//AC Asymmetric Supercapacitor

In a two-electrode cell containing 3M KOH as an electrolyte, an asymmetric supercapacitor (ASC) was constructed. The NiO/Ni aerogel and AC were adapted as the positive and negative electrodes, respectively. The charge storage between the positive and negative electrodes was balanced using the following equation:

(2) $$\frac{m_{+}}{m_{-}}=\frac{C_{s-}\;∆V_{-}}{C_{s+}\;∆V_{+}}$$

where subscripts “+” and “−“ are the positive and negative electrodes, respectively.

The mass of AC obtained using the abovementioned method was mixed with carbon black (10 wt%) and PVDF (5 wt%), emulsified with NMP solution, and then mixed with an agate motor to form a uniform slurry. Finally, the as-prepared slurry was dried at 60 °C for 12 h in a vacuum oven and marked as negative electrodes in the hybrid device.

### 2.6. Calculation of Specific Capacitance: ED and PD

Three-electrode and two-electrode measurements were obtained using 3M KOH, and CV studies were conducted at selected scan rates between 1 and 200 mV/s for 0 to 0.6 V vs. Ag/AgCl (saturated with KCl). The GCD curves were obtained at the same potential window in the current density range of 1 to 15 A/g for the three-electrode measurements and at 0 to 1.6 V for current densities ranging from 1 to 5 A/g for the two-electrode measurements, i.e., the NiO/Ni/NF and AC/NF electrodes, respectively.

The specific capacitance and specific capacity for the two- and three-electrode arrangements were calculated using Equation (1). The ED and PD of NiO/Ni/NF were calculated using the following equations:

(3) $$E=\frac{C_{s}\times ∆V^{2}}{7200}$$

(4) $$P=\frac{3600\times E}{∆t}$$

where *E* and *P* are the energy (Wh/kg) and power (W/kg) densities for the two-electrode device, respectively; Δ*V* is the voltage window (V); *C* is the specific capacitance (F/g); and Δ*t* is the discharge time (s).

## 3. Results and Discussion

**Figure 1:**
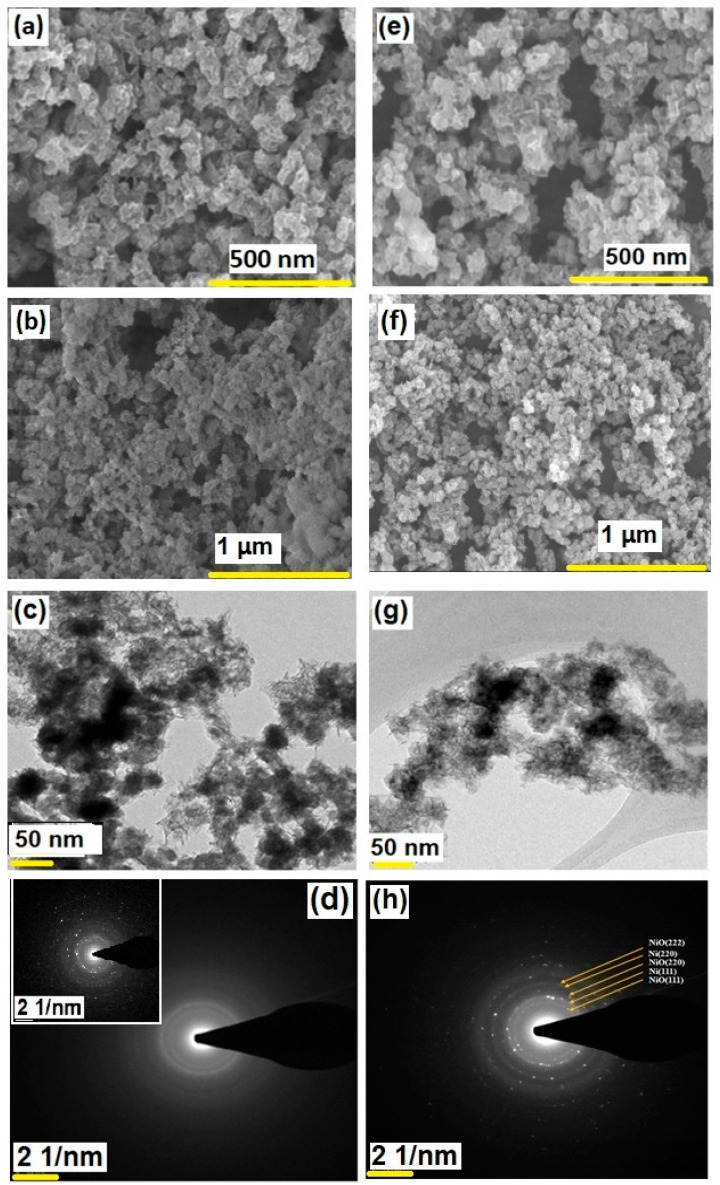
(**a**,**b**) FESEM images of α-Ni(OH)_2_ and (**e**,**f**) NiO/Ni, HRTEM images of (**c**) α-Ni(OH)_2_ and (**g**) NiO/Ni, and corresponding SAED patterns of (**d**) α-Ni(OH)_2_ and (**h**) NiO/Ni. (Inset in figure (**d**) shows the SAED pattern of α-Ni(OH)_2_).

The SEM images (Figure 1) of the as-prepared nickel aerogel [α-Ni(OH)_2_] and annealed samples (NiO/Ni) showed flaky nanoporous waferlike nanoflower structures. The α-Ni(OH)_2_ samples showed significantly high aggregation, as observed from the TEM studies, and when annealed, the resultant NiO/Ni samples showed more open structures with less aggregation. The TEM images of the samples also confirmed the presence of nanoflakes with edge widths ranging from 1 to 2 nm with aggregated nickel nanoparticles inside the flaky nanoflower structures. The SAED (selected area diffraction pattern) pattern shown in Figure 1h also confirmed the presence of crystallite sites, and the d-spacing indicated the presence of the (111), (220), and (222) crystal planes of NiO and the (111) and (220) crystal planes of Ni. The SAED pattern of the as-synthesized aerogel in Figure 1d also showed the presence of clouded rings, indicating the amorphous nature of α-Ni(OH)_2_. The inset in Figure 1d shows some crystalline nature, but mostly the aerogels were amorphous in general. Aerogels are generally known to be amorphous solids, but noble metal aerogels have shown polycrystalline nature; moreover, the transition metal aerogels when heated to high temperatures show less amorphous and more crystalline features. The polycrystalline nature arises due to the aggregation of a large number of crystallite held together by thin layers of amorphous solid. In spite of their polycrystalline nature, the NiO/Ni aerogels exhibited continuous nano flaky structure without undergoing any structural degradation. The EDS and elemental constitution of the NiO/Ni aerogels are given in Figures S1 and S2a–c of Supplementary Materials. The TEM-EDS analysis shows the presence of Ni and O, indicating the constituents of the aerogel; the Cu in the EDS is from the grid used for TEM analysis.

**Figure 2:**
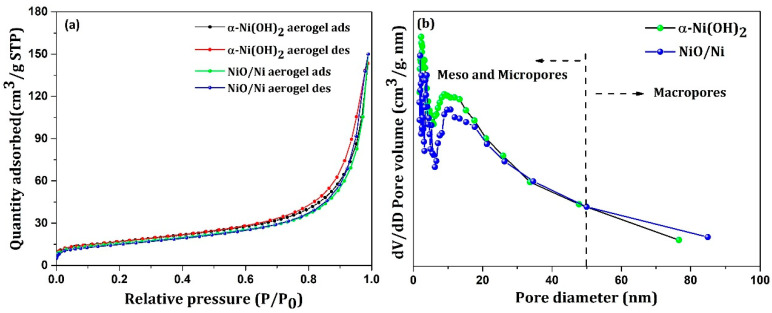
(**a**) N_2_ adsorption–desorption isotherms and (**b**) corresponding pore size distribution plots of α-Ni(OH)_2_ and NiO/Ni.

The surface area and porous nature of the aerogels were investigated by conducting nitrogen sorption tests. Figure 2a,b show the N_2_ adsorption–desorption isotherms of the α-Ni(OH)_2_ and NiO/Ni aerogels, respectively, as well as the corresponding pore diameter and pore volume plots. The nature of the isotherms for both samples was similar with similar surface area values of 54.8 m^2^/g and 55.6 m^2^/g, respectively. The volume of adsorption was also noted to be similar, and adsorption followed a type IV isotherm with an H1 hysteresis loop denoting the existence of both mesopores and macropores in both aerogel samples. As shown in Figure 2b, the majority of pore sizes ranged from 10 to 50 nm, indicating the mesoporous nature of both aerogels, with some macroporous (>50 nm) region. Porosity is an important criterion in determining the capacity of any electrode material because small pores present more active sites for ion accumulation. Therefore, the high surface area and porosity values of both aerogel samples indicate good electrochemical capacity.

**Figure 3:**
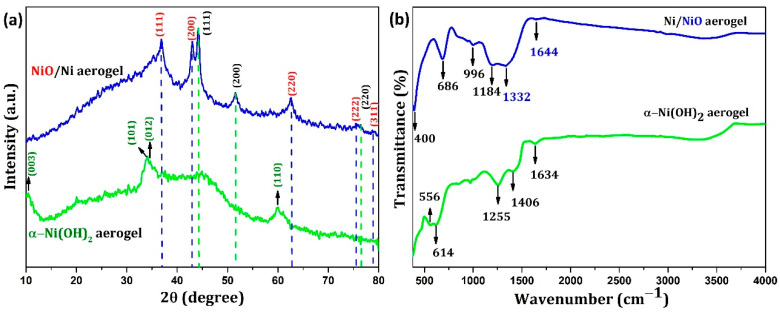
(**a**) XRD patterns and (**b**) FTIR spectra of the α-Ni(OH)_2_ and NiO/Ni aerogels.

XRD patterns of the as-synthesized aerogels and annealed (400 °C) samples indicated the presence of α-Ni(OH)_2_ and NiO/Ni, respectively, as shown in Figure 3a. The peaks at 2θ values of 10.8°, 33.7°, 34.3°, and 59.7° can be indexed to the (003), (101), (012), and (110) planes (JCPDS card no. 38-0715), respectively, indicating that α-Ni(OH)_2_ has a single-phase rhombohedral crystal structure. It is known from the literature [33] that as the temperature increases to 250 °C, α-Ni(OH)_2_ starts to transform into NiO with (111), (200), and (220) planes. The XRD measurement of our annealed sample showed predominant peaks at 2θ values of 36.8°, 43.1°, and 62.7°, corresponding to the (111), (200), and (220) major planes of NiO, respectively. Other minor planes were also observed for NiO at 2θ values of 75.5° and 78.8°, corresponding to the (222) and (311) planes of NiO, respectively (JCPDS card no. 47-1049). The peaks for Ni were observed at 2θ values of 44.1°, 51.5°, and 76.4° (minor peak) corresponding to the (111), (200), and (220) planes of nickel, respectively (JCPDS card no. 03-1051) [34,35].

The FTIR spectra of the α-Ni(OH)_2_ and NiO/Ni aerogel samples are shown in Figure 3b. The spectrum highlighted in green shows typical characteristics of α-Ni(OH)_2_. The FTIR spectrum of the α-Ni(OH)_2_ aerogel shows a broad band at approximately 3200–3700 cm^−1^, indicating the stretching vibrations of hydroxyl groups having hydrogen bonding with H_2_O, and the peak at 1634 cm^−1^ can be assigned to the water bending mode. For the annealed samples, i.e., NiO/Ni aerogels, the peaks were observed at 3250–3700 cm^−1^ in the form of a broad peak indicating the stretching vibrations of the hydroxyl groups’ stretching region; the other peaks were observed at 1644, 1332, 1184, 996, 686, and 400 cm^−1^. The peaks at 996 and 686 cm^−1^ correspond to the distinctive modes of the Bunsenite NiO phase [35].

**Figure 4:**
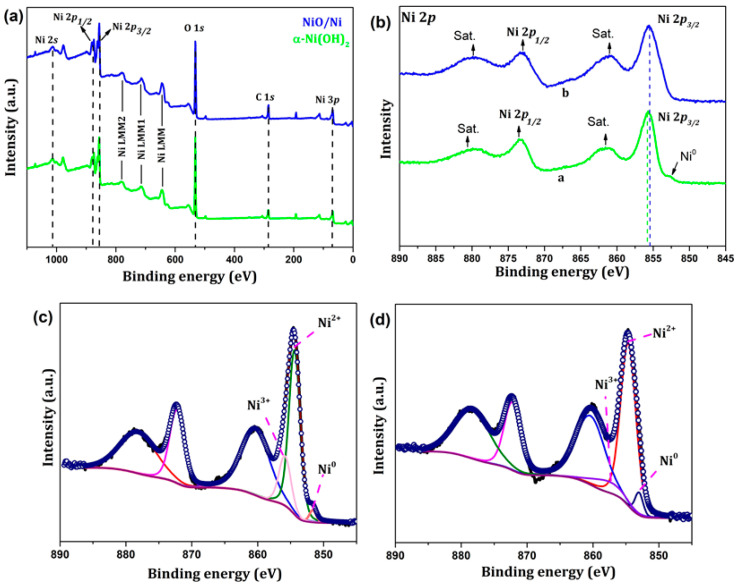
XPS spectra of the (**a**) α-Ni(OH)_2_ and (**b**) NiO/Ni aerogel samples, and the deconvoluted spectra (**c**,**d**) of the Ni 2p_3/2_ and Ni 2p_1/2_ peaks.

XPS spectra were obtained to determine the chemical composition and valence state of each element in the as-synthesized and annealed aerogels. Figure 4 shows the XPS profiles of α-Ni(OH)_2_ and NiO/Ni. Both XPS spectra show high quantities of O and Ni, and trace amounts of C. The C 1 s peak at 284.4 eV may be due to the CO_2_ absorbed into the aerogel samples exposed to air and adventitious hydrocarbons generated with the instrument itself [36]. A general observation is that due to its structural nature, α-Ni(OH)_2_ is always in the hydrated form because water is intrinsic to its structure. For comparison purposes, the XPS of α-Ni(OH)_2_ and NiO/Ni deconvoluted fitting is given in Figure S3 of the Supplementary Materials. The deconvoluted spectra for Ni 2p_3/2_ and Ni 2p_1/2_ peaks are given in Figure 4c,d.

As shown in Figure 4, the peaks at 855.7 and 873.4 eV corresponded to the Ni 2p_3/2_ and Ni 2p_1/2_ of nickel hydroxide, respectively, and the broad peaks at 854–856 eV and 872–874 eV of the annealed samples corresponded to the Ni 2p_3/2_ and Ni 2p_1/2_ of Ni^3+^, respectively, which also may carry information about the Ni^2+^, Ni^3+^, and Ni^0^ states when deconvoluted [37]. This might be possible if there is some surface Ni_2_O_3_ present for the annealed samples. Two broad satellite peaks were observed at 859–863 eV and 877–881 eV in both the as-synthesized and annealed samples. A distinctive Ni^0^ peak was present in α-Ni(OH)_2_ at 852.5 eV, which was merged with the Ni 2p peaks in the NiO/Ni aerogel samples. The presence of a minute amount of metallic nickel could have arisen from the high temperature oxidation reaction in the presence of oxygen and trace carbon. Additionally, the oxide layer became predominant with annealing, and this is consistent with results reported by Du et al. [38]. Two vibrational satellite peaks appearing at 862.2 and 879.5 eV correspond to Ni 2p_3/2_, which shifted negatively in the case of the annealed NiO/Ni aerogel samples. The O 1 s peak at 531.3 eV indicates the presence of Ni-O bonds, and the slight hump in the annealed NiO/Ni aerogel samples shows the presence of NiOOH. The XPS results provided a plethora of valuable information about the chemical structure of the aerogel samples and indicated the as-synthesized aerogels as possible candidates for energy storage applications.

**Figure 5:**
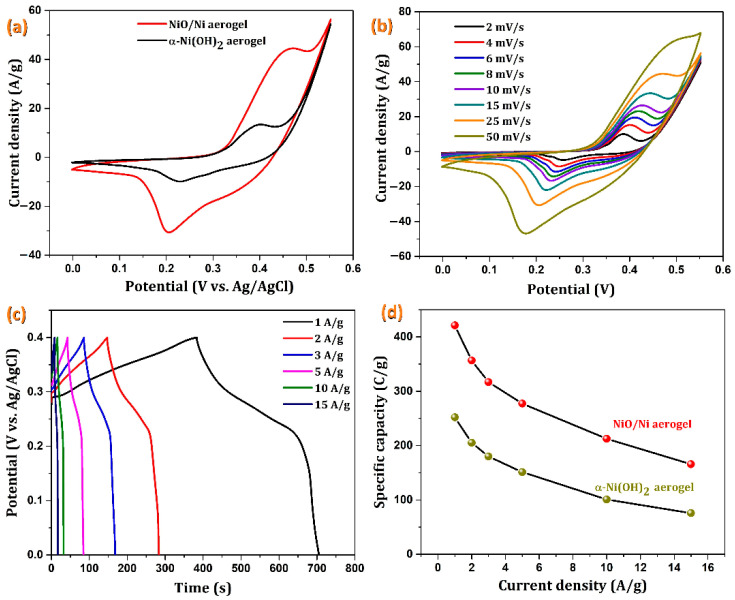
(**a**) CVs of the α-Ni(OH)_2_- and NiO/Ni-modified nickel foam electrodes, (**b**) dependence of the scan rate of the NiO/Ni electrode, (**c**) GCD of the NiO/Ni/NF electrode, and (**d**) plot of the specific capacity vs. the current densities of the NiO/Ni/NF electrodes.

The electrochemical behaviors of the as-synthesized and annealed samples were studied using CV, GCD, and EIS. The comparative electrochemical performance of the three-electrode studies for α-Ni(OH)_2_ and NiO/Ni was carried out using CV at a scan rate of 25 mV/s in the potential range of 0 to 0.55 V vs. Ag/AgCl, as shown in Figure 5a. The large area under the CV curves indicates a higher charge storage capacity of NiO/Ni than that of the α-Ni(OH)_2_ aerogel. Hence, the suitability of the NiO/Ni samples as supercapacitor electrodes was further studied at current densities ranging from 4 to 50 mV/s. As expected, the annealed aerogel samples exhibited a clear redox behavior at different scan rates, indicating their pseudocapacitive nature. The redox reactions expected in the active materials on the electrode surface are as follows:

(5) $$\alpha -\mathrm{Ni}{\left(\mathrm{OH}\right)}_{2}+{\mathrm{OH}}^{-}\frac{\mathrm{Charge}}{\mathrm{Discharge}}\;\gamma -\mathrm{NiOOH}+H_{2}O+e^{-}$$

(6) $$\mathrm{NiO}+{\mathrm{OH}}^{-}\frac{\mathrm{Charge}}{\mathrm{Discharge}}\;\gamma -\mathrm{NiOOH}+e^{-}\;$$

The redox reactions occurring in the α-Ni(OH)_2_ and NiO/Ni aerogel samples are expressed in Equations (5) and (6), respectively. The transformation of the “α” form of Ni(OH)_2_ to the “γ” form takes place with a reversible exchange of 1.7 e per Ni atom with a theoretical capacity of 390 mAh/g [39], whereas the theoretical capacity of NiO [40] is approximately 718 mAh/g. The cyclic voltammograms clearly show that the potential difference between the redox peaks is consistent with the properties of a Faradaic pseudocapacitive material. The redox peaks in the CV are due to the conversion of Ni^2+^ and Ni^3+^, which also involves the insertion and extraction of OH^−^ ions. The shape of the CV and the increase in current density in the CVs with scan rates are also indicative of the fast redox process occurring in the electrode. An increase in the redox peaks was observed as scan rates increased, which could be attributed to the increased polarization. Although the morphologies of the as-synthesized and annealed samples appear to be similar, the activity and, in turn, the capacity of the annealed samples (NiO/Ni) appear to be far higher than that of α-Ni(OH)_2_ [41].

Next, GCD studies were carried out for the as-synthesized and annealed samples in the presence of various mass-normalized current densities with a 0~0.4 V potential range, as shown in Figure 5c. Because nickel hydroxide behaves like a battery material, the charge–discharge curves show distinct potential plateaus analogous to battery-type materials. The iR (potential drop due to solution resistance) drop of the NiO/Ni aerogel electrodes is much lower than ~0.01 V at 1 A/g and increases to ~0.02 V at 15 A/g. Hence, such low ohmic drop values signify a better electrode performance. The plateau may be caused by the diffusion of hydroxyl ions to the surface region from the bulk phase of the active material and the presence of different phases [42]. Therefore, these materials undergo bulk redox reactions. The nonlinearity in the charge–discharge curves is also indicative of the battery-type nature of the electrode material. This occurs because of the combined contribution of the redox reaction and electrochemical adsorption at the electrode–electrolyte interface. In the specific capacity vs. current density plot, as shown in Figure 5d, the annealed aerogel (NiO/Ni) samples showed higher specific capacity values than did similar materials reported in the literature. The comparison of GCD results for α-Ni(OH)_2_ and NiO/Ni is given in Figure S4 of the Supplementary Materials.

The specific capacitance and specific capacity of the NiO/Ni aerogel samples based on the galvanostatic discharge curves were calculated to be 1060, 890, 795, 680, 520, and 430 F/g and 422, 356, 316, 276, 212, and 164 C/g at current densities of 1, 2, 3, 5, 10, and 15 A/g, respectively. Figure 5d shows the variation in capacity with current densities for the annealed NiO/Ni aerogel samples. At high current densities, the charge compensating OH^−^ becomes slower, resulting in the observed decrease in capacity. The lowest current density dictates the actual specific capacity of the material. In our study, we observed a higher capacity contribution of NiO/Ni aerogels than the α-Ni(OH)_2_ aerogels, but the behavior was not similar in nanoparticle morphologies [43]. This could be due to the participation of the finer aerogel structures toward capacity when compared to the micro- or nanoparticle morphologies of the same metal ions.

**Figure 6:**
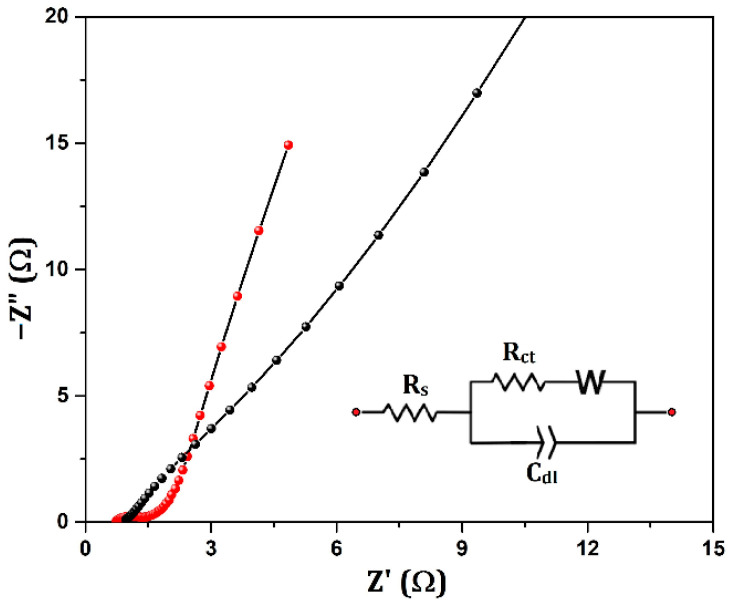
EIS plot of the NF (black) and NiO/Ni/NF(red) electrode at open circuit voltage (OCV) (the inset is the equivalent circuit representation).

The comparison of various aerogel- and metal oxide-based supercapacitor electrodes and their performances are provided in Table 1. EIS studies were also performed to further verify the suitability of the active material for energy applications. The EIS was carried out, and the measured R_s_ and R_ct_ values from the Nyquist plots NiO/Ni (1 Ω and 1.4 Ω) show that the lower values of annealed sample are suitable for delivering high capacities. In the NiO/Ni aerogel samples, we observed a limiting resistance R_L_ (0.4 Ω) at intermediate frequencies. This is a slight Warburg impedance behavior occurring at 45° from the horizontal axis, after which the line becomes almost vertical, indicating good capacity values. This behavior was observed in NiO on activated carbon electrodes, with steep slopes for the Warburg impedance, and the impedance data could be fitted with a modified Randles circuit consisting of two resistors, two capacitors, and a Warburg element [44]. In Figure 6, the equivalent circuit representation is shown as an inset of the Nyquist plot of NiO/Ni in the three-electrode arrangement.

**Figure 7:**
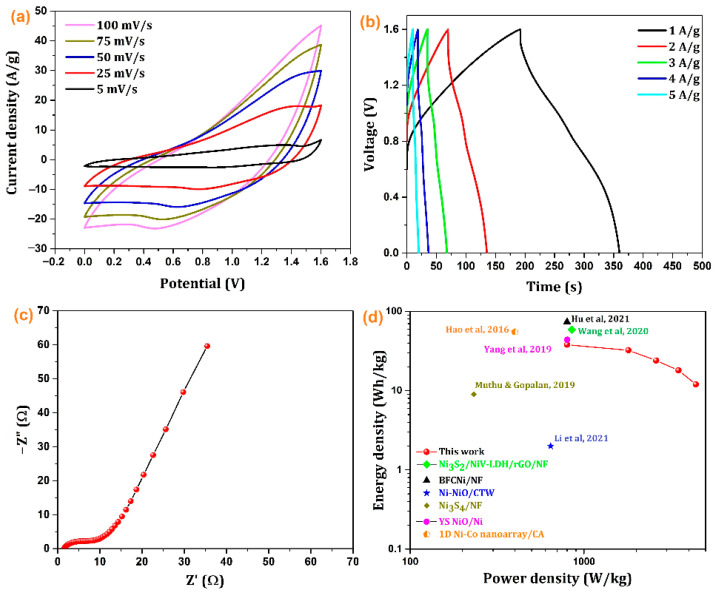
(**a**) CV of the device at scan rates of 5 to 100 mV/s, (**b**) GCD for the device at current densities of 1 to 5 A/g, (**c**) Nyquist plot of the device at OCV, and (**d**) Ragone plot (ED vs. PD curves) for the device at various current densities [6,7,8,9,10,11].

The practical applications of the NiO/Ni supercapacitor electrode were investigated by constructing an asymmetric device with an NiO/Ni-positive electrode and an AC-negative electrode, the masses of which were balanced using Equation (2). Figure 7 shows the electrochemical performance of the device. The potential window was fixed from 0 to 1.6 V based on the CV measurements to ensure that there was no oxygen or hydrogen evolution at the electrodes. CV profiles were obtained at various scan rates of 5, 25, 50, 75, and 100 mV/s. The voltammograms showed typical behavior of a two-electrode device with a redox-active material and AC. Next, to explore the electrochemical properties of the assembled device, the GCD measurements were obtained at different current densities. Based on the GCD measurements, the assembled device delivered a specific capacity of 169, 131, 101, 73, and 50 F/g at 1, 2, 3, 4, and 5 A/g, respectively. The GCD curves were symmetrical, indicating the excellent rate-delivering properties of the device [6]. In addition, EIS measurements were obtained to assess the changes in the resistances and capacitance behavior of the material. R_s_ remained almost the same, and R_ct_ increased slightly from 2 to 10 Ω. The Warburg behavior at intermediate frequencies and diffusion-limited behavior (straight line) at low frequencies was not significantly altered. The practical application of other similar aerogel-based electrode materials is given in Table 2.

The device performance is highly affected by the energy and power density. The Ragone plot in Figure 7d shows the comparative energy PD of the NiO-based asymmetric supercapacitor device. The assembled device delivers an ED of 38 Wh/kg at a PD of 800 W/kg. The values of PD-ED obtained in this study were compared with those reported for similar electrode materials [6,7,8,9,10,11]. The ED and PD of the device were higher than those of the other NiO-based devices in the literature, such as BFCNi/NF [7] (BFCNi: bamboo fiber-derived carbon, nickel-based hydroxide) that showed a PD of 799 W/kg and an ED of 74 Wh/kg, Ni_3_S_2_/NiV-LDH/rGO/NF//AC [6] (Ni_3_S_2_/NiV-LDH/rGO: nickel sulfide/nickel vanadium-layered double hydroxide/reduced graphene oxide composite) that showed an ED of 59 Wh/kg and a PD of 852 W/kg, Ni-NiO/CTW//CTW HSC [8] that achieved a PD of 643 W/kg and an ED of 2 Wh/kg, a Ni_3_S_4_/NF [9] symmetric device that showed an ED of 9 Wh/kg with a PD of 233 W/kg, and YS NiO/Ni [10] (YS: Yolk-shelled) that achieved an ED of 44 Wh/kg and a PD of 801 W/kg. A 1D Ni-Co oxide and sulfide nanoarray/carbon aerogel [11] hybrid asymmetric capacitor yielded a PD of 400 W/kg at energy densities of 55 and 48 Wh/kg.

**Figure 8:**
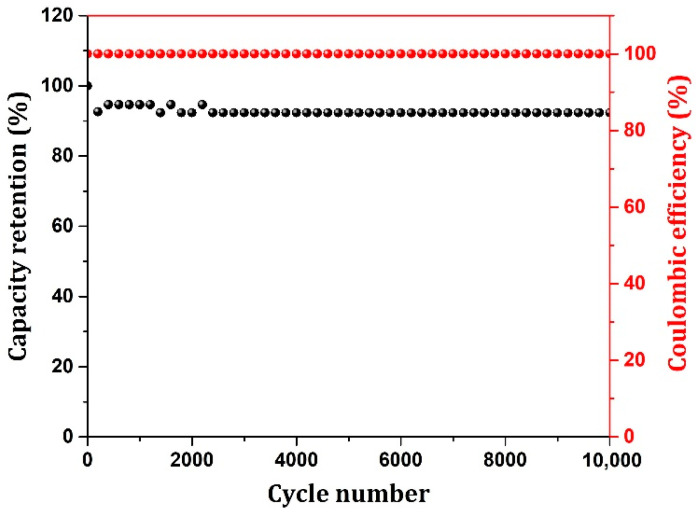
Coulombic efficiency and capacity retention of the device (NiO/Ni//AC).

Figure 8 shows the columbic efficiency and capacitance retention for the NiO/Ni aerogel electrode up to 10,000 cycles. The ED and PD values obtained for our device are excellent in comparison to other similar materials and aerogel-based devices. The device also showed high cycling stability up to 10,000 cycles with a capacity retention of 92%, demonstrating the excellent and reliable efficiency of the electrode materials. Exhibiting light-weight, flexible properties and excellent capacitance with stability, our aerogel materials will be competitive in cutting-edge energy storage device applications in the near future.

## 4. Conclusions

In summary, α-Ni(OH)_2_ and NiO/Ni aerogels were synthesized using a one-step sol-gel reduction method. The α-Ni(OH)_2_ and annealed NiO/Ni aerogels exhibited and retained a flaky nanoflower morphology, with good porosity and uniformity. This is the first study on the conversion of α-Ni(OH)_2_ aerogels to NiO/Ni aerogels through annealing at 400 °C in an autoclave. The specific capacity of the annealed NiO/Ni aerogel was more than twice that of the α-Ni(OH)_2_ aerogel precursor. The specific capacity of the NiO/Ni/NF aerogels for the three-electrode and two-electrode systems were 1060 F/g and 169 F/g at a current density of 1 A/g, respectively. The ED and PD of the device were found to be excellent at 38 Wh/kg and 800 W/kg with a better cycling stability up to 10,000 cycles. This indicates the outstanding electrochemical performance of the active electrode material for practical two-electrode applications. The present synthesis strategy of the electrode materials and aerogel morphology will enable ultralightweight supercapacitors to meet the increasing energy demands in various applications.

## Figures and Tables

**Table 1:** Comparison of various aerogel-based supercapacitor electrodes and their performances.

System	Capacitance Calculation Method	Capacitance (F/g)/ Capacity(C/g)	Electrolyte	Reference
NiO aerogel	CV	797 (10 mV/s)	6M KOH	[45]
Carbon aerogel/Ni	GCD	181 (1 mA/cm^2^)	6M KOH	[46]
Ni foam/Graphene aerogel	GCD	366 (2 A/g)	6M KOH	[47]
Nano Ni-doped carbon aerogel	GCD	110 (1 A/g)	6M KOH	[48]
rGO and Ni(OH)_2_	GCD	561 (2 A/g)	1M KOH	[49]
NiO/Graphene aerogel	GCD	587 (1 A/g)	6M KOH	[50]
NiO NPs	CV	549 (1 mV/s)	1M KOH	[51]
NiO NPs/CC	CV	132 (5 mV/s)	1M KOH	[52]
NiFeP@NiCo_2_S_4_	GCD	1602/720 (10 A/g)	2M KOH	[53]
Ni-ZnS	CV	191/131 (10 mV/s)	2M KOH	[54]
NiO/Ni/NF	GCD	1060/422 (1 A/g)	3M KOH	This Study

**Table 2:** The practical application of aerogel-based electrode materials and their performance comparison.

System	Capacitance (F/g)	Capacity Retention (Number of Cycles)	Reference
MnO_2_ aerogel	139 @ 1 A/g	93% (5000)	[55]
NiS/NiO	91 @ 1 A/g	93% (30,000)	[56]
Graphene aerogel/CeO_2_	156 @ 1 A/g	91% (4000)	[57]
rGO/RuO_2_ aerogels	310 @ 1 A/g	83% (5000)	[58]
Cobalt sulfide aerogel	72 @ 1 A/g	-	[59]
NiO/Ni/NF	169 @ 1 A/g	92% (10,000)	This Study

## Data Availability

Not applicable.

## Supplementary Materials

The following are available online at https://www.mdpi.com/article/10.3390/nano12213813/s1: Figure S1: TEM-EDS of the NiO/Ni aerogel indicating the presence of Ni and O. The Cu peak arises from the grid for TEM analysis; Figure S2: (a) Elemental images from TEM-EDS proving the presence of (b) Ni, (c) O of NiO/Ni aerogel indicating the presence of the two elements. The Cu peak arises from the grid used for TEM analysis; Figure S3: O1s XPS spectra of α-Ni(OH)_2_ and NiO/Ni and their deconvolution are also included; Figure S4. GCD of α-Ni(OH)_2_-and NiO/Ni-modified nickel foam electrodes at 1 A/g current density given for comparison between the two electrodes.
