# Supplementary material for: NiO/Ni Nanowafer Aerogel Electrodes for High Performance Supercapacitors
Source: Nanomaterials (Basel). 2022 Oct 28;12(21):3813. doi: 10.3390/nano12213813 (PMC9655204; doi:10.3390/nano12213813)
Supplement: Supplementary file 1 [file nanomaterials-12-03813-s001.zip › nanomaterials-1975574-supplementary.pdf]

Supplementary materials

NiO/Ni Nanowafer Aerogel Electrodes for High Performance Supercapacitors

Ramya Ramkumar, Ganesh Dhakal, Jae-Jin Shim and Woo Kyoung Kim *

School of Chemical Engineering, Yeungnam University, Gyeongsan 38541, Korea
* Correspondence: author: wkim@ynu.ac.kr

.

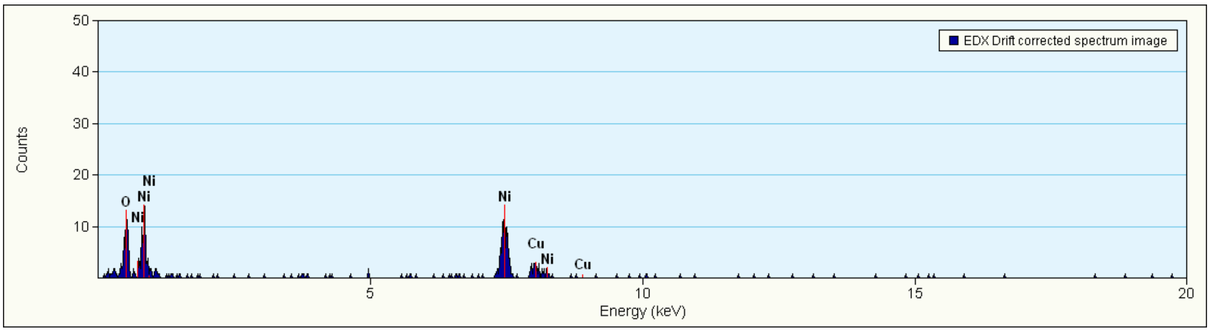

Figure S1. TEM-EDS of NiO/Ni aerogel indicating the presence of Ni and O. The Cu peak arises from the grid for TEM analysis.

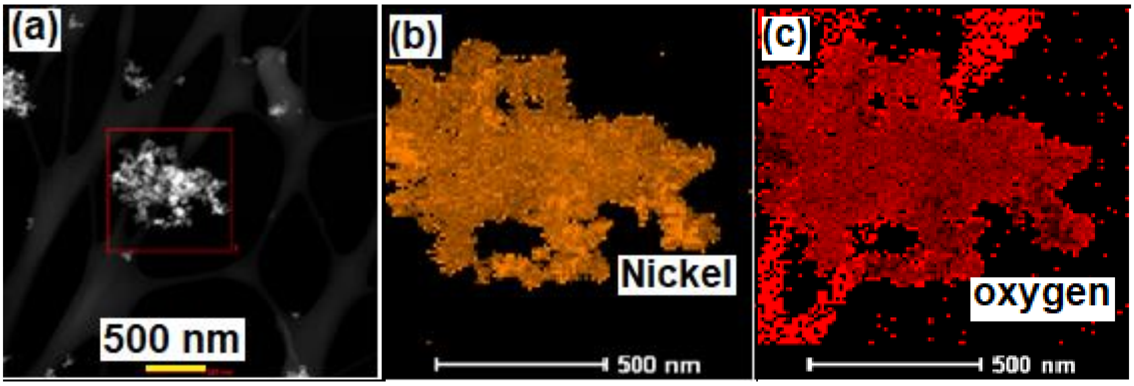

Figure S2. (**a**) Elemental images from TEM-EDS proving the presence of (**b**) Ni, (**c**) O of NiO/Ni aerogel indicating the presence of the two elements. The Cu peak arises from the grid used for TEM analysis.

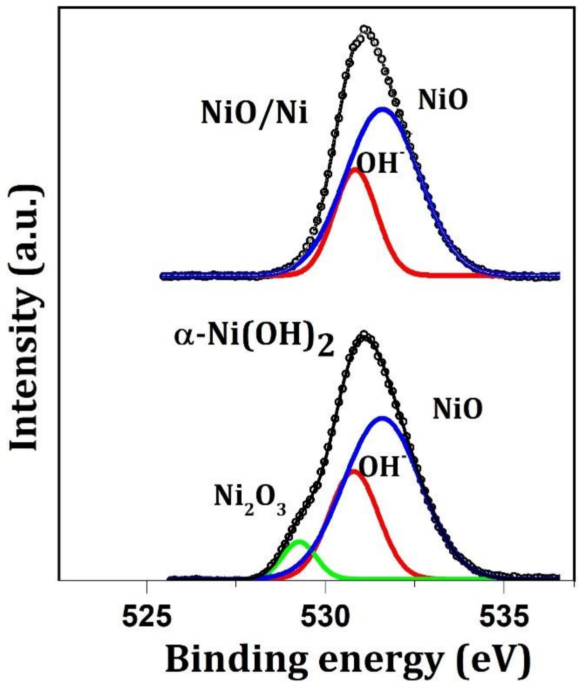

Figure S3. O1s XPS spectra of α-Ni(OH)$_2$ and NiO/Ni and their deconvolution is also included.

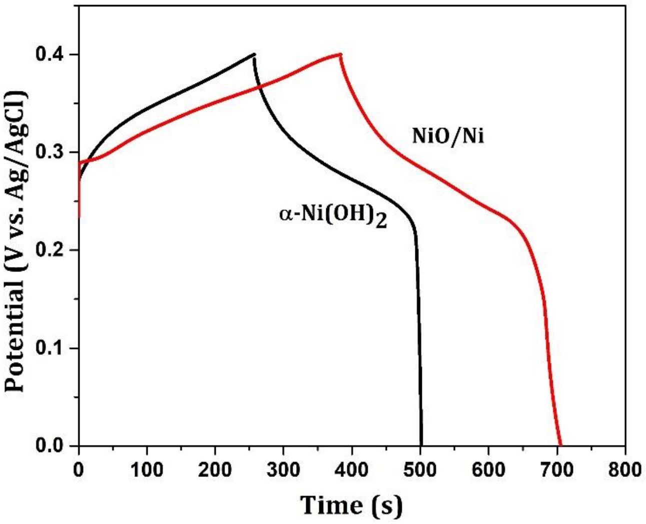

Figure S4. GCD of α-Ni(OH)$_2$-and NiO/Ni-modified nickel foam electrodes at 1 A/g current density given for comparison between the two electrodes materials.
